# RNA-Rocket: an RNA-Seq analysis resource for infectious disease research

**DOI:** 10.1093/bioinformatics/btv002

**Published:** 2015-01-07

**Authors:** Andrew S. Warren, Cristina Aurrecoechea, Brian Brunk, Prerak Desai, Scott Emrich, Gloria I. Giraldo-Calderón, Omar Harb, Deborah Hix, Daniel Lawson, Dustin Machi, Chunhong Mao, Michael McClelland, Eric Nordberg, Maulik Shukla, Leslie B. Vosshall, Alice R. Wattam, Rebecca Will, Hyun Seung Yoo, Bruno Sobral

**Affiliations:** ^1^Virginia Bioinformatics Institute, Virginia Tech, Blacksburg, VA 24060, USA, ^2^Center for Tropical & Emerging Global Diseases, University of Georgia, Athens, GA 30602, USA, ^3^Penn Center for Bioinformatics and ^4^Department of Biology, University of Pennsylvania, Philadelphia, PA 19104, USA, ^5^European Bioinformatics Institute (EMBL-EBI), Wellcome Trust Genome Campus, Hinxton, CB10 1SD, UK, ^6^Department of Biological Sciences, University of Notre Dame, Notre Dame, IN 46556, USA, ^7^Department of Computer Science and Engineering, University of Notre Dame, Notre Dame, IN 46556, USA, ^8^Eck Institute for Global Health, University of Notre Dame, Notre Dame, IN 46656-0369, USA, ^9^University of California, Department of Microbiology and Molecular Genetics, Irvine, California, USA and ^10^The Rockefeller University, Howard Hughes Medical Institute, New York, NY 10065, USA

## Abstract

**Motivation:** RNA-Seq is a method for profiling transcription using high-throughput sequencing and is an important component of many research projects that wish to study transcript isoforms, condition specific expression and transcriptional structure. The methods, tools and technologies used to perform RNA-Seq analysis continue to change, creating a bioinformatics challenge for researchers who wish to exploit these data. Resources that bring together genomic data, analysis tools, educational material and computational infrastructure can minimize the overhead required of life science researchers.

**Results:** RNA-Rocket is a free service that provides access to RNA-Seq and ChIP-Seq analysis tools for studying infectious diseases. The site makes available thousands of pre-indexed genomes, their annotations and the ability to stream results to the bioinformatics resources VectorBase, EuPathDB and PATRIC. The site also provides a combination of experimental data and metadata, examples of pre-computed analysis, step-by-step guides and a user interface designed to enable both novice and experienced users of RNA-Seq data.

**Availability and implementation:** RNA-Rocket is available at rnaseq.pathogenportal.org. Source code for this project can be found at github.com/cidvbi/PathogenPortal.

**Contact:** anwarren@vt.edu

**Supplementary information:** Supplementary materials are available at *Bioinformatics* online.

## 1 Introduction

Transcriptomic analysis using high-throughput sequencing continues to increase in popularity due to low sequencing costs, its sensitivity, reproducibility and its ability to sample the entire transcriptome (Wang *et al*., 2009). As an active area of research, there are many variations of RNA-Seq protocols, data types and tools that continue to evolve. As a result, there is no ‘one size fits all’ solution for doing RNA-Seq analysis. The range of considerations facing life scientists who want to leverage this technology can demand significant investment of time and resources. For this reason, we have created RNA-Rocket, an RNA-Seq analysis service that enables infectious disease research for prokaryotic and eukaryotic pathogens as well as vectors and host genomes.

RNA-Rocket is built on Galaxy (Blankenberg *et al*., 2001; Giardine *et al*., 2005; Goecks *et al*., 2010), with modifications to help simplify the process for routine use and provide a guided user experience. RNA-Rocket integrates data from the PATRIC, EuPathDB and VectorBase Bioinformatics Resource Centers (BRCs) and is provided by Pathogen Portal (pathogenportal.org), a resource linking all BRCs funded by the National Institute of Allergy and Infectious Diseases (NIAID).

The RNA-Rocket service leverages multiple open source software tools to provide a free resource where users can upload their RNA-Seq data, align them against a genome and generate quantitative transcript profiles. This service also provides streaming of alignment and annotation results back to the appropriate BRC so that users can view results in the relevant BRC, using annotations and tools provided in support of transcriptomic analysis.

The *Pat*hosystems *R*esource *I*ntegration *C*enter (PATRIC) is the all-bacteria Bioinformatics Resource Center (patricbrc.org) (Wattam *et al*., 2013). PATRIC provides researchers with an online resource that stores and integrates a variety of data types (e.g. genomics, transcriptomics, protein-protein interactions, 3-D protein structures and sequence typing data) along with any associated metadata. The eukaryotic pathogen databases (EuPathDB: eupathdb.org) provide access to a variety of data types from important human and veterinary parasites such as *Plasmodium* (malaria), *Cryptosporidium* (cryptosporidiosis) and kinetoplastida (i.e. *Trypanosoma brucei* and *Leishmania* species.) (Aurrecoechea *et al*., 2013). VectorBase (vectorbase.org) is a bioinformatics resource for invertebrate vectors of human parasites and pathogens (Megy *et al*., 2012). It currently hosts the genomes of 35 organisms including mosquitoes (20 of which are *Anopheles* species), tsetse flies, ticks, lice, kissing bugs and sandflies.

## 2 Implementation

RNA-Rocket takes advantage of many different open-source projects to enable users to upload and analyze their own data. We use the Galaxy system to consolidate and provide the tools and services necessary to process high-throughput sequencing data. The use of Galaxy has many benefits: showing provenance information for data creation, including the tools and parameters used to process data; support for batch analysis for multiple samples; providing a mechanism for results sharing across research groups and publishing for external references such as presentations or publications and its integration of tools and projects in the larger bioinformatics community.

Before users can run analysis on the RNA-Rocket site they must first upload their data in FASTQ format. Using standard Galaxy interfaces RNA-Rocket supports upload via URL, FTP, HTTP and direct transfer via the European Nucleotide Archive (Leinonen *et al*., 2011), which can be searched using ENA, SRA, GEO and ArrayExpress identifiers. To enable basic RNA-Seq processing, RNA-Rocket provides users with a set of pre-determined Galaxy workflows configured to use existing BRC genomes and annotations. The primary workflow for RNA-Seq analysis aligns short read data to a reference genome using Bowtie2 (Langmead and Salzberg, 2012) or TopHat2 (Kim *et al*., 2013), assembles transcripts using Cufflinks, and generates coverage bedGraph and BigWig files using BEDTools (Quinlan and Hall, 2010) and UCSC tools (Kuhn *et al*., 2013), respectively. This workflow generates BAM files and tab-delimited output, which can be used to determine transcript structure and the level of expression in the target organism.

The site also provides the ability to conduct differential expression analysis using Cuffdiff, part of the Cufflinks suite and the ability to visualize data generated using CummeRbund (Trapnell *et al*., 2012). When users submit their jobs to RNA-Rocket, they are queued and run on a first-come, first-served basis on a compute cluster using a modern, high-density computer architecture.

**Fig. 1.**
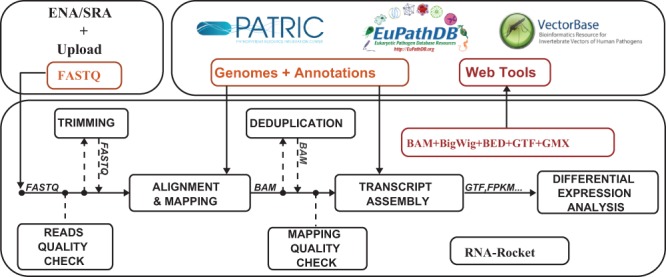
Data flow in RNA-Rocket

The site features an interactive concept diagram, which highlights the appropriate processing step(s), based on the concept that the user is interested in. Clicking on a concept diagram component gives information about the corresponding processing steps that fulfill the component (Fig. 1). To assist users, the site provides a ‘Launch Pad’ menu system that breaks down the context and rationale for executing a particular step, the input required from the user and the output that is generated.

RNA-Rocket is designed to make users aware of three major concerns: the quality base calls in their sequencing reads, the number of reads aligned from their sample and accounting for PCR bias. Base call quality can vary depending on the sequencing technology and sample preparation. Poor quality sequencing can impact the certainty with which a read can be mapped to the genome (Li *et al*., 2008). Although, modern aligners endeavor to account for Phred quality scores when performing alignment, it is important for users to be aware that low quality input can lead to low levels of alignment. To help users account for this, RNA-Rocket provides access to the FastQC tool (http://www.bioinformatics.babraham.ac.uk/projects/fastqc) for determining the quality profile of sample reads, as well as Sickle (https://github.com/najoshi/sickle) and Trimmomatic (Bolger *et al*., 2014), which can be used to automatically trim off low quality base calls from the ends of sequencing reads. Once alignment is performed the user also has the option to check the quality profile and number of reads mapped using a modified version of the SAMStat tool (Lassmann *et al*., 2011). The RNA-Rocket site highlights these quality control steps in both the concept diagram and interactive menu system. These three options enable the researcher to maximize the amount of their sample being used through iterative pre-processing, alignment and evaluation.

RNA-Rocket also provides a quality control option for removing PCR bias that can occur as a result of library preparation (Aird *et al*., 2011). Certain sequences may be overrepresented if their composition leads to bias in the amplification process (Benjamini and Speed, 2012). For paired-end data this option uses Picard tools (http://picard.sourceforge.net/) to ‘collapse’ multiple read pairs that have identical coordinates for the first and second read into a single representative pair.

In addition to RNA-Seq analysis, RNA-Rocket also supports ChIP-Seq (Chromatin Immunoprecipitation-Sequencing) analysis by providing access to the peak calling program MACS (Zhang *et al*., 2008). After mapping the reads to the reference genome, a user may use MACS to identify and quantify the ChIP signal enriched genomic regions. The mapped reads and genome coverage can be directly viewed via the BRC genome browsers and the peak calling result can be viewed and downloaded via the RNA-Rocket web interface.

The RNA-Rocket site is updated daily with genomes and annotations from each of the contributing BRCs. Thousands of genomes are organized and indexed using Bowtie2 (Langmead and Salzberg, 2012) and SAMtools (Li *et al*., 2009) to enable alignment and bias correction for abundance estimation, respectively. Reference resources are organized by BRC so that results can be streamed and analyzed within the context of the data provider.

## 3 Results

The RNA-Rocket project modifies the existing Galaxy code so that Galaxy workflows can be constructed in advance by system administrators and shared to users through a tiered menu system. This menu system, referred to as ‘Launch Pad’, organizes RNA-Seq processing steps conceptually and gives increasing detail as the user progress towards launching a job, i.e. an analysis step. This system also asks the user to populate their project space, known as a ‘history’ in Galaxy terms, with the necessary files before attempting to configure the parameters for their job. This is designed to minimize confusion when attempting to setup an analysis and promote organization for projects involving many files and processing steps. Using the workflow system, RNA-Rocket provides pre-formulated solutions for common problems that users encounter. These workflows are easily adapted to new tools and are publicly available for download to enable offline analysis and customization for researchers.

RNA-Rocket provides example data from each of the BRC projects so that users can familiarize themselves with the site using real data. Some of these data are provided through the Driving Biological Project (DBP) initiative, research projects competitively enabled through NIAID’s BRC program designed to drive innovation at the BRC sites based on the needs of the research community. Each dataset is provided as both ‘before’ and ‘after’ project spaces so that users can import the project into their own user space, run their own analysis and view pre-existing results. See the Supplementary Material for more details on this data.

By combining experimental concepts with file-based requirements, the user interface aims to guide life science researchers through the process of RNA-Seq data analysis while making them aware of quality control caveats. After results have been computed at the RNA-Rocket site they can be streamed back to the respective BRC depending on the reference organism selected for analysis. This provides users with the ability to process and analyze their RNA-Seq data remotely without having to download potentially large files to their own computer.

RNA-Rocket is a free service that can be used by life-science researchers to process and analyze their RNA-Seq data. The site maintains up-to-date genome and annotation data through NIAD’s Bioinformatic Resource Centers. By leveraging BRC data and the Galaxy system, the RNA-Rocket project can provide up-to-date tools and capability despite the rapidly changing landscape of RNA-Seq analysis.

## Supplementary Material

Supplementary Data
