# Supplementary material for: RNA-Rocket: an RNA-Seq analysis resource for infectious disease research
Source: Bioinformatics. 2015 Jan 7;31(9):1496–8. doi: 10.1093/bioinformatics/btv002 (PMC4410666; doi:10.1093/bioinformatics/btv002)
Supplement: Supplementary Data [file supp_31_9_1496__index.html]

RNA-Rocket: An RNA-Seq Analysis Resource for Infectious Disease Research — RNA-Rocket: an RNA-Seq analysis resource for infectious disease research — RNA-Rocket: an RNA-Seq analysis resource for infectious disease research — Supplementary Data 

# RNA-Rocket: an RNA-Seq analysis resource for infectious disease research

## Supplementary Data

files

**Files in this Data Supplement:**

- Supplementary Data - docx file
