# Supplementary material for: RNA-Rocket: an RNA-Seq analysis resource for infectious disease research
Source: Bioinformatics. 2015 Jan 7;31(9):1496–8. doi: 10.1093/bioinformatics/btv002 (PMC4410666; doi:10.1093/bioinformatics/btv002)
Supplement: Supplementary Data [file supp_btv002_supplementary_material_rnarocket.docx]

Brought online in early 2012 RNA-Rocket has steadily increased its user base, the amount of data submitted and generated, and the number of genomes supported and analyzed. As of 12/2014 the site has over 975 registered users, 9Tb of uploaded data and 5Tb of output generated. Multiple research projects have leveraged the free compute resources to process their transcriptomic data (Lew et al., 2013; Stringer et al., 2014) and support continues to expand as requests come in for additional genomes and annotations.

## More About NIAID BRC’s

S1.1 PATRIC

At PATRIC data types are summarized for individual genomes and across taxonomic levels, and also for individual genes. All genomes in PATRIC, currently more than 21,000, are consistently annotated using Rapid Annotations using Subsystems Technology, RAST (Overbeek et al., 2013). Comparisons of PATRIC and NCBI (Pruitt, Tatusova, Klimke, & Maglott, 2009) annotations are available. PATRIC provides a variety of ways for researchers to find data of interest and a private workspace where they can store both genomic and gene associations, and their own private data, including RNA-Seq data downloaded from RNA-Rocket. Both private and public data can be analyzed together using a suite of tools to perform comparative genomic or transcriptomic analysis.

S1.2 EuPathDB

A graphical search system enables users to interrogate underlying data such as genomic sequences, annotation, functional genomic data (i.e., RNA-Seq and proteomic) and clinical data (Fischer et al., 2011). Users of EuPathDB can construct complex search strategies such as asking for genes that contain predicted secretory signal peptides that are expressed during a specific developmental stage, that are absent from mammals and contain a large number of polymorphisms. Results of searches may be viewed or downloaded. In addition, users may upload their own data such as RNA or DNA sequence coverage plots into a genome browser (GMOD), where they can view their uploaded data in the context of their genome of interest with any data already available in EuPathDB (Stein et al., 2002). Data generated through RNA-Rocket are conveniently linked to the genome browser in EuPathDB sites for rapid visualization.

S1.3 VectorBase

VectorBase has also recently incorporated *Biomphalaria glabrata*, the snail intermediate host of schistosomiasis and the house fly, *Musca domestica*. Hosted data range from genomic features and expression data to population genetics and ontologies. Updates to VectorBase (or releases) occur every two months; both public and private data can be queried or analyzed with a variety of available tools including BLAST and Expression and Population Biology browsers (Megy et al., 2012). The overarching goal of VectorBase resource is to support “-omics” aided efforts to improve or develop new vector control strategies.

**Table S1**Number of pre-indexed genomes with annotations and streaming support

| Type | Count |
| --- | --- |
| Host | 11 |
| PATRIC | 21648 |
| EuPathDB | 157 |
| VectorBase | 35 |

Counts taken 12/2014. Host genomes and their annotations are obtained from the UCSC Genome Browser Database (Karolchik et al., 2014)

## RNA-Rocket as an educational tool

The RNA-Rocket resource has been, and continues to be, used in numerous scientific workshops as a way to familiarize researchers with processing RNA-Seq experiments. Example project spaces are provided as a starting point for users to explore RNA-Seq analysis. These project spaces were also created in conjunction with How-To documents, available at http://rnaseq.pathogenportal.org/page/list_published, that take the user through the step-by-step process of generating output for analysis. We briefly describe the design and intent of each experiment represented in the example data as a preliminary for further user exploration.

S2.1 PATRIC educational data

RNA-Rocket makes available transcriptome data generated through a PATRIC Driving Biological Project for *Salmonella typhimurium* 14028. Non-typhoidal *Salmonella* (NTS) are widespread and common human pathogens, causing tens of millions of infections and hundreds of thousands of fatalities annually worldwide, especially among young and elderly victims with co-morbidities. These data seek to characterize the transcriptome of *S. typhimurium* in a wide range of conditions to provide both basal reference profiles for future comparison and experimental evidence for improving annotation. Nine different growth conditions were used: rich media (LB) logarithmic and stationary phase growth; rich media at pH 5.5 logarithmic phase growth; M9 minimal media logarithmic and stationary phase growth; M9 minimal media containing glucose logarithmic and stationary phase growth; and early stationary growth with and without nitric oxide, which has been implicated in host defense. All experiments were performed twice on different days to account for biological and technical variation. Sequencing is paired-end and strand specific using Illumina encoding 1.5 and 1.9. This data provides users with examples for examining expression profiles, doing differential expression analysis, and viewing feature annotations computed from RNA-Seq.

S2.2 EuPathDB educational data

EuPathDB has utilized RNA-Rocket as an educational tool to empower bench scientists to perform their own RNA-Seq analysis without the need to master command line scripting. To this end EuPathDB includes a module on RNA-Seq analysis using RNA-Rocket in all of its workshops. Workshop participants learn about RNA-Seq file format and the tools used to align and analyze RNA-Seq data. In addition, they work with a sample dataset available from SRA (Leinonen, Sugawara, & Shumway, 2011), setup TopHat and Cufflinks parameters and kickoff the workflow. The results of the workflow are examined including a description of output file formats and conversion between formats. Students then transfer their data to the genome browser in EuPathDB to visualize the data in the context of the genome, annotation and other available functional data. EuPathDB has also developed YouTube tutorials that describe how to use RNA-Rocket, which can be found, along with the workshop module, at the RNA-Rocket site.

To enable users to explore data associated with a eukaryotic pathogen RNA-Rocket provides short read sequences, alignments, and assembled transcripts for *Encephalitozoon cuniculi* (Grisdale, Bowers, Didier, & Fast, 2013), a parasitic organism known to infect humans with compromised immunity due to HIV-infection. Grisdale et al. generated RNA-Seq data for *E. cuniculi* at three time-points: 24 hr, 48 hr, and 72 hr post-infection of rabbit kidney fibroblast, RK13 cells. Short read sequences were obtained from SRA.

S2.3 VectorBase educational data

The transcriptome of *Aedes aegypti* sampled from neural tissues, and other tissues involved in host-seeking behavior, is profiled in Illumina short read data generated through a VectorBase DBP for this dengue and yellow fever vector. The resulting neurotranscriptome (BioProject accession PRJNA236239 and ID 236239) is a catalogue of all mosquito genes expressed in the central nervous system and head sensory appendages that are thought to be involved in host-seeking behavior; the antenna and rostrum (maxillary palp and proboscis); and the brain, which directs the sensory-motor behavior required for a blood meal. Other tissues included ovaries, which are affected by blood feeding, and the mosquito legs and abdominal tips (ovipositors), known to carry chemosensory sensilla involved in gustatory behaviors such as oviposition. Beyond comparing sex and tissue-specific differences in gene expression, this project sought to examine tissue specific gene expression changes associated with blood-feeding state, and such changes differed between two strains of *A. aegypti* with divergent host preferences. This transcriptome contains 147 SRA experiments; data provided on the RNA-Rocket site contains one library for the brain tissue of females fed with sugar. At VectorBase users are able to compare alignments and features computed in this experiment against a variety of other transcriptomic experiments for *Aedes aegypti*.

## BRC Analysis

S3.1 PATRIC

When using RNA-Rocket, novel transcript prediction can be performed in two different ways: assemble transcripts using an existing annotation and assemble transcripts using alignment information only. When assembling transcripts using only short read alignments, from reads with strand information, resulting predictions more closely resemble the operon structure for bacterial data. This can provide information on the polycistronic structure of mRNA.

PATRIC supports three major data types generated by RNA-Seq analysis at RNA-Rocket through their corresponding file formats: alignments (BAM and bigWig), transcript assembly (GTF), and differential expression analysis data (GeneMatrix file). RNA-Seq data can be streamed directly from RNA-Rocket to the appropriate organism’s genome browser, JBrowse (Skinner, Uzilov, Stein, Mungall, & Holmes, 2009), at PATRIC, allowing researchers to examine these data and compare it with the annotations that are provided for each genome. By streaming the transcript assembly results from RNA-Rocket to PATRIC a user can determine novel genomic features predicted by their RNA-Seq experiment relative to existing PATRIC and NCBI annotations using PATRIC's 'Combination Track' ability. When using RNA-Rocket, novel transcript prediction can be performed in two different ways: assemble transcripts using an existing annotation and assemble transcripts using alignment information only. When creating a combination track from transcripts assembled using an existing annotation, a user can employ PATRIC's 'subtract features' option to easily see all genomic features predicted by the RNA-Seq experiment that are not present in the annotation. When using transcripts assembled without previous annotations, resulting predictions more closely resemble the operon structure from a given bacteria. In this case a combination track can be created using PATRIC's 'subtract coverage' feature to view all those regions, which were part of a transcribed region of the genome but not in an existing annotation. This allows users to easily encapsulate and view novel predictions made by their RNA-Seq experiment. These combination tracks can then be downloaded as a GFF3, BED, or Sequin table file. As seen in Figure S2, PATRIC’s browser also allows viewing of alignment BAM files, to show alignment details, and BigWig to see an overall expression profile.

To support differential gene expression analysis, PATRIC provides a suite of integrated tools to explore, visualize, analyze and compare published and private datasets using a secure workspace. Researchers can upload their own pre-processed transcriptomics datasets, generated by either RNA-Rocket or a microarray experiment to their private workspace and analyze them with data analysis and visualization tools available at PATRIC. Metadata-driven filters facilitate searching for experiments of interest; dynamic filterable gene lists, a heat map, and clustering tools allow researchers to quickly find genes that are up or down regulated in the select experimental conditions or have similar expression patterns across one or more conditions. PATRIC’s pathway summary tool summarizes metabolic pathways related to selected genes. As of July 2014, the PATRIC database included 807 transcriptomics experiments across 72 bacterial genera, each incorporated with manually curated data and metadata, all available for comparison. Filtering on experimental metadata can help researchers locate similar experiments to those in their private data and subsequently look for similarities in up- or down-regulation of genes.


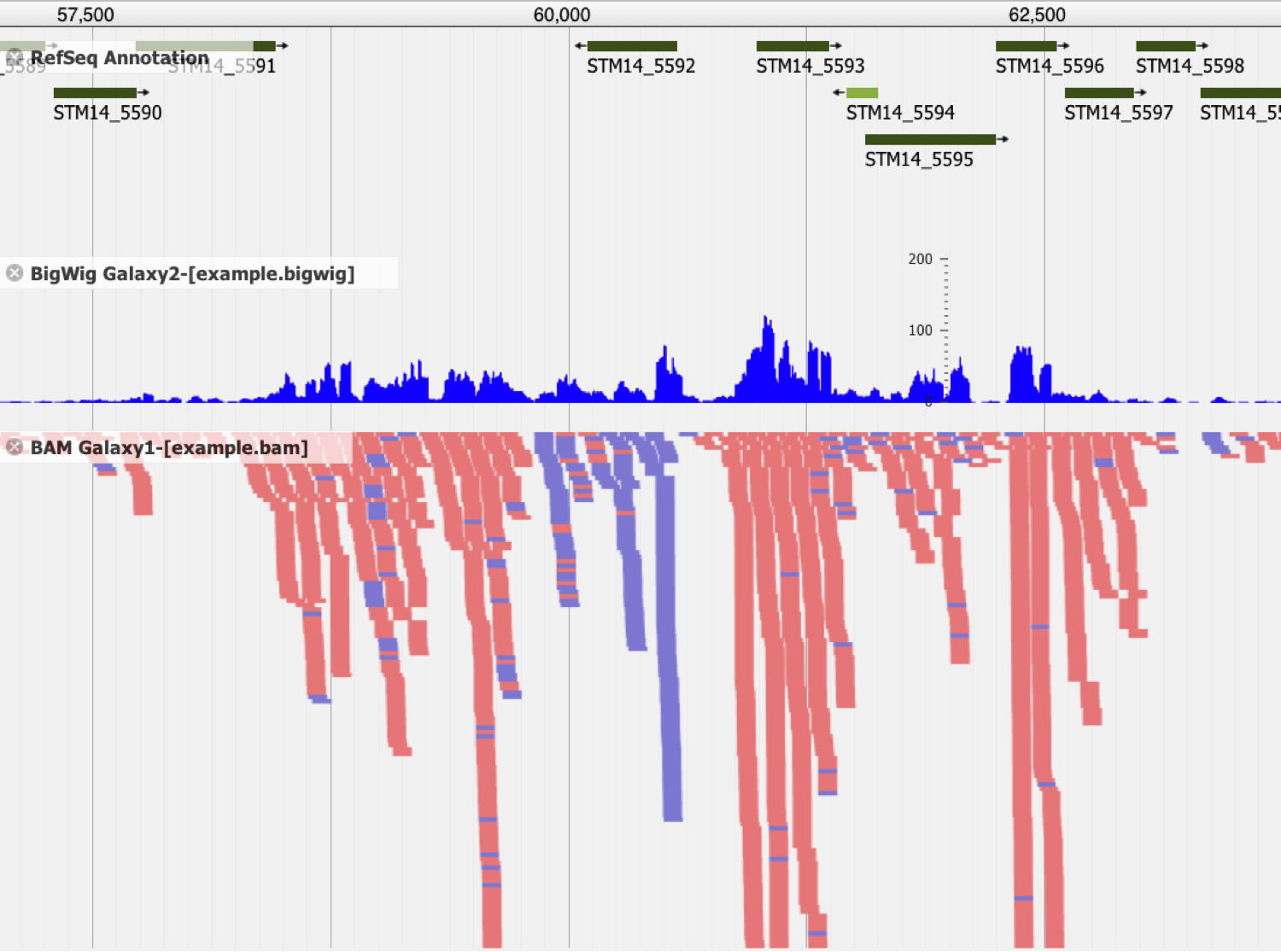


Fig. S2 PATRIC view of existing annotation (top) with a BigWig expression profile (middle) and BAM alignment view (bottom)

S3.2 EuPathDB

Results computed on RNA-Rocket for eukaryotic pathogens provide links for streaming to the corresponding EuPathDB page for the organism under analysis. This support extends to all the divisions of EuPathDB including AmoebaDB, CryptoDB, GlardiaDB, PiroplasmaDB, PlasmoDB, ToxoDB, TrichDB, TriTrypDB, and the pan-fungal genome resource FungiDB. EuPathDB supports streaming of RNA-Rocket results using BAM, GTF, and bigWig files through GBrowse. As seen in Figure S3, at EuPathDB a user can view predicted exons and transcripts given by Cufflinks and the expression profile given by BAM and bigWig files computed from short read alignment by TopHat. This enables users to see experimental results alongside EuPathDB annotations. Custom tracks are associated with a users login to make them available between sessions and the snapshot functionality allows users to save combinations of tracks of interest.

*
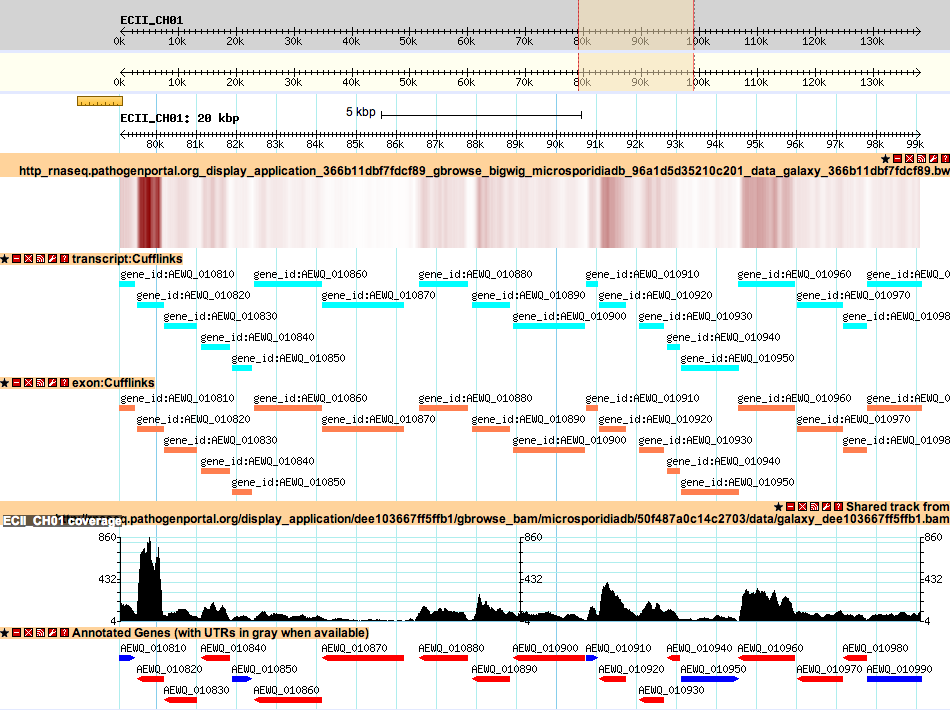
*

Fig. S3 EuPathDB view of RNA-seq data using GBrowse. From top to bottom tracks include nucleotide scale, bigWig density, GTF predicted transcripts and genes, BAM expression view, and EuPathDB annotations

S3.3 VectorBase

RNA-Rocket supports streaming for 35 different vector genome assemblies from VectorBase. Tracks can be streamed for BigWig files that enable the user to see the expression profile and GTF files which show predicted transcript structure. VectorBase uses the Ensembl browser and supports the ability of users to save custom tracks in association with their user account. For many of the supported organisms, VectorBase provides pre-computed transcriptomic experiments that can be enabled to view alongside a user’s RNA-Seq data. Figure S4 illustrates streaming of the example *A. aegypti* data for brain tissue of females fed with sugar alongside an enabled track that represents a pre-computed result from a female *A. aegypti* brain post-bloodmeal. These data are easily compared to existing VectorBase annotations for protein-coding genes and non-coding RNA to identify potentially undiscovered genomic features.

*
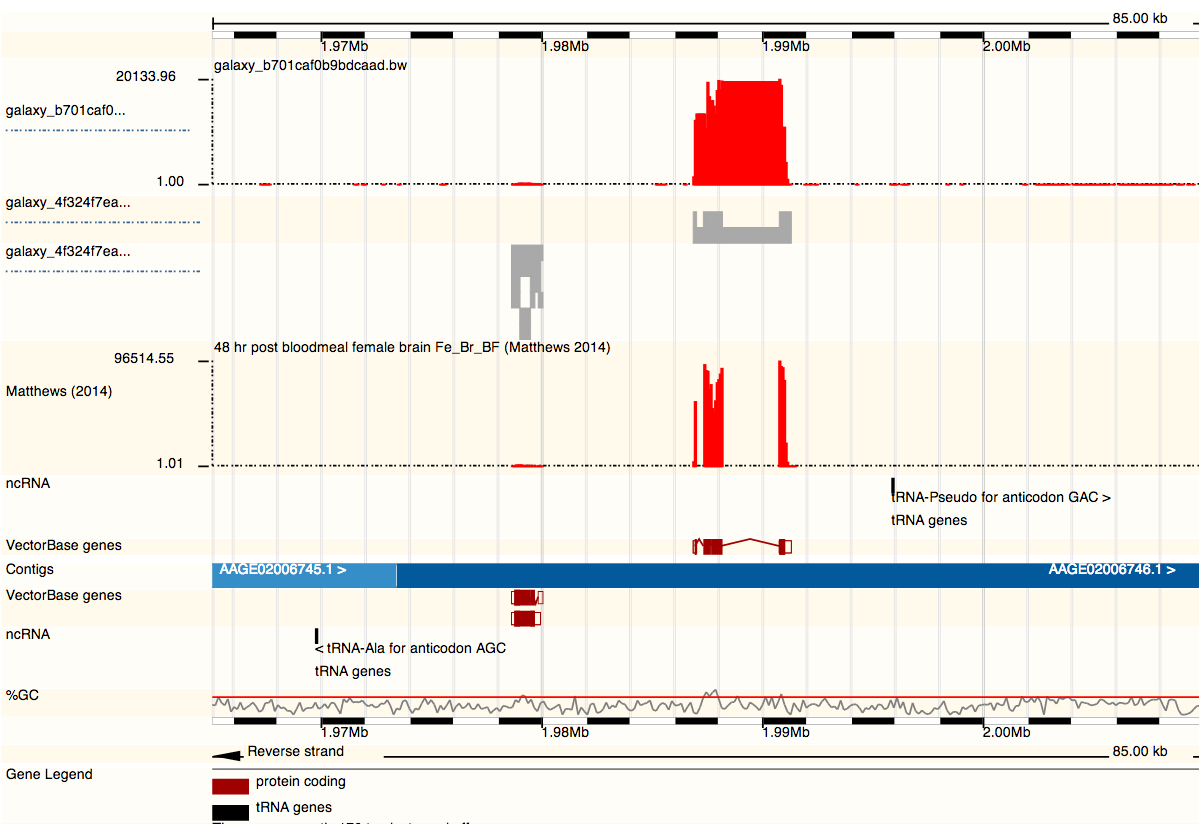
*

Fig. S4 VectorBase view of (top down) bigWig expression profile, GTF RNA-Seq predictions, a pre-computed VectorBase experiment, and VectorBase annotations

**References**

Fischer, S., Aurrecoechea, C., Brunk, B. P., Gao, X., Harb, O. S., Kraemer, E. T., . . . Stoeckert, C. J. (2011). The Strategies WDK: a graphical search interface and web development kit for functional genomics databases. *Database (Oxford), 2011*, bar027. doi: 10.1093/database/bar027

Grisdale, C. J., Bowers, L. C., Didier, E. S., & Fast, N. M. (2013). Transcriptome analysis of the parasite Encephalitozoon cuniculi: an in-depth examination of pre-mRNA splicing in a reduced eukaryote. *BMC Genomics, 14*, 207. doi: 10.1186/1471-2164-14-207

Karolchik, D., Barber, G. P., Casper, J., Clawson, H., Cline, M. S., Diekhans, M., . . . Kent, W. J. (2014). The UCSC Genome Browser database: 2014 update. *Nucleic Acids Res, 42*(Database issue), D764-770. doi: 10.1093/nar/gkt1168

Leinonen, R., Sugawara, H., & Shumway, M. (2011). The sequence read archive. *Nucleic Acids Res, 39*(Database issue), D19-21. doi: 10.1093/nar/gkq1019

Lew, J. M., Mao, C., Shukla, M., Warren, A., Will, R., Kuznetsov, D., . . . Sobral, B. (2013). Database resources for the tuberculosis community. *Tuberculosis (Edinb), 93*(1), 12-17. doi: 10.1016/j.tube.2012.11.003

Megy, K., Emrich, S. J., Lawson, D., Campbell, D., Dialynas, E., Hughes, D. S., . . . Wilson, D. (2012). VectorBase: improvements to a bioinformatics resource for invertebrate vector genomics. *Nucleic Acids Res, 40*(Database issue), D729-734. doi: 10.1093/nar/gkr1089

Overbeek, R., Olson, R., Pusch, G. D., Olsen, G. J., Davis, J. J., Disz, T., . . . Stevens, R. (2013). The SEED and the Rapid Annotation of microbial genomes using Subsystems Technology (RAST). *Nucleic Acids Res*. doi: 10.1093/nar/gkt1226

Pruitt, K. D., Tatusova, T., Klimke, W., & Maglott, D. R. (2009). NCBI Reference Sequences: current status, policy and new initiatives. *Nucleic Acids Res, 37*(Database issue), D32-36. doi: 10.1093/nar/gkn721

Skinner, M. E., Uzilov, A. V., Stein, L. D., Mungall, C. J., & Holmes, I. H. (2009). JBrowse: a next-generation genome browser. *Genome Research, 19*(9), 1630-1638. doi: 10.1101/gr.094607.109

Stein, L. D., Mungall, C., Shu, S., Caudy, M., Mangone, M., Day, A., . . . Lewis, S. (2002). The generic genome browser: a building block for a model organism system database. *Genome Research, 12*(10), 1599-1610. doi: 10.1101/gr.403602

Stringer, A. M., Currenti, S., Bonocora, R. P., Baranowski, C., Petrone, B. L., Palumbo, M. J., . . . Wade, J. T. (2014). Genome-scale analyses of Escherichia coli and Salmonella enterica AraC reveal noncanonical targets and an expanded core regulon. *J Bacteriol, 196*(3), 660-671. doi: 10.1128/JB.01007-13
